# Prospective randomised phase II study of gemcitabine at standard or fixed dose rate schedule in unresectable hepatocellular carcinoma

**DOI:** 10.1038/sj.bjc.6601369

**Published:** 2003-11-11

**Authors:** Z Guan, Y Wang, S Maoleekoonpairoj, Z Chen, W S Kim, V Ratanatharathorn, W H H Reece, T W Kim, M Lehnert

**Affiliations:** 1 Tumor Hospital of Sun Yat-Sen University, Medical Sciences, East Dongfeng Road, Guangzhou 510060, PR China; 2 Shanghai Changhai Hospital, The 2nd Military Medical University, No. 174, Changhai Road, Shanghai 200433, PR China; 3 Pra Mongkutklao Hospital of the Royal Thai Army, 315 Rajavithi Road, Bangkok 10400, Thailand; 4 Fuzhou General Hospital, No. 156, Xi Huan Bei Road, Fuzhou, Fujiang 350025, PR China; 5 Samsung Medical Center, Sung Kyun Kwan University School of Medicine, 50, Ilwon-Dong, Kangnam-Gu, Seoul 135-710, Korea; 6 Ramathibodi Hospital, Rama VI Road, Bangkok 10400, Thailand; 7 Clinical Outcomes and Research Institute, Eli Lilly Australia, Level 1/16 Giffnock Ave., Macquarie Park, NSW 2113, Australia; 8 Asan Medical Center, 388-1 Poong Nap-Dong, Song Pa-Gu, Seoul 138-736, Korea; 9 Eli Lilly Asian Operations, Limited, 27th Floor, CITIC Tower, 1 Tim Mei Avenue, Central, Hong Kong, SAR China

**Keywords:** hepatocellular carcinoma, gemcitabine

## Abstract

The present randomised phase II study was an effort to evaluate single-agent gemcitabine as a first-line systemic treatment of Asian patients with unresectable hepatocellular carcinoma (HCC). Gemcitabine was given via intravenous infusion at 1250 mg m^−2^ on days 1 and 8 of 3-week cycles. Patients were randomised to receive gemcitabine as a 30-min intravenous infusion (standard schedule) or at a fixed dose rate (FDR) of 10 mg m^−2^ min^−1^. A total of 50 patients were enrolled in the study, of whom 48 received study therapy. One patient on standard schedule had a partial response, for an overall response rate of 2.1% (95% CI: 0.05–11.1%). The median time to progression and survival time were 46 and 97 days, respectively. The overall rates of Grade 3 or 4 haematological and nonhaematological toxicities were 39.6 and 64.6%, respectively, with no significant difference between the two treatment arms. There were no drug-related deaths and severe clinical toxicities were rare. Both schedules of gemcitabine were safe and toxicity was well manageable in this patient population. However, gemcitabine seems no more active than other cytotoxic agents when used alone for systemic treatment of advanced HCC.

Hepatocellular carcinoma (HCC) is a major cause of cancer death, in Asia and worldwide (Pisani *et al*, 1999). Most patients have inoperable disease at the time of diagnosis and need systemic therapy at some point of their disease. No systemic therapy has shown reproducible benefit in controlled clinical trials and treatment outcome has remained poor (Venook, 1994; Simonetti *et al*, 1997; Fong *et al*, 2001).

The antimetabolite gemcitabine (GEMZAR®) is a deoxycytidine analogue (2′,2′-difluorodeoxycytidine, dFdC) that inhibits DNA synthesis (Plunkett *et al*, 1989). Preclinical studies of gemcitabine have shown promising activity in a human HCC model (Graziadei *et al*, 1998). A phase II study of single-agent gemcitabine in advanced HCC in Taiwan found a response rate of 17.8% and good tolerance (Yang *et al*, 2000). In that study, gemcitabine was given as an intravenous infusion over 30 min, which is the standard mode of gemcitabine administration. Preclinical and clinical studies have found that the intracellular accumulation of dFdC triphosphate, the active moiety of gemcitabine incorporated into DNA, gets saturated at gemcitabine levels of 15–20 *μ*mol l^−1^ and can be maximised by the administration of gemcitabine at a fixed dose rate (FDR) of 10 mg m^−2^ min^−1^ (Grunewald *et al*, 1990, 1991; Abbruzzese *et al*, 1991; Tempero *et al*, 1999). In two phase I studies, the maximum tolerated dose of gemcitabine at FDR was found to be 1500 mg m^−2^ (Brand *et al*, 1997) and 1800 mg m^−2^ (Touroutoglou *et al*, 1998). Both studies suggested a phase II starting dose of 1500 mg m^−2^. The toxicity profile of FDR gemcitabine was similar to that of the standard schedule, with granulocytopaenia and thrombocytopaenia the dose-limiting toxicities (Brand *et al*, 1997; Touroutoglou *et al*, 1998). A randomised phase II study in pancreatic cancer has suggested that FDR gemcitabine may produce higher efficacy than standard schedule (Tempero *et al*, 1999). The present multinational randomised phase II study was an effort to confirm the previously observed activity of standard schedule gemcitabine in Asian patients with unresectable HCC and to evaluate the activity and toxicity of FDR gemcitabine in this patient population.

## PATIENTS AND METHODS

Eligible patients had a tissue diagnosis of HCC or serum alpha-fetoprotein of at least 400 ng l^−1^ plus liver imaging studies that were considered highly suggestive of HCC. Patients had to have distant metastases (stage IV) or locally advanced disease (stage IIIB) not eligible for curative surgery, and bidimensionally measurable disease. Prior systemic chemotherapy was not allowed. Intra-arterial chemotherapy was allowed if given more than 3 months prior to enrolment and not including more than one cytotoxic drug. Other inclusion criteria included: Karnofsky performance status (KPS) of 70 or higher and estimated life expectancy of at least 12 weeks; age of at least 18 years and no higher than 75 years; white blood cell count ⩾3.0 × 10^9^ l^−1^, absolute neutrophil count (ANC) ⩾2.0 × 10^9^ l^−1^, platelets ⩾75 × 10^9^ l^−1^, haemoglobin ⩾9.0 g dl^−1^; total serum bilirubin ⩽4 × upper limit of normal (ULN), ALT and AST ⩽4 × ULN, and serum albumin ⩾20 g l^−1^; serum creatinine ⩽1.5 × ULN; Okuda stage I or II (Okuda *et al*, 1985); and lastly, written informed consent. Exclusion criteria included: central nervous system metastases; any other concomitant tumour therapy; pregnancy or breastfeeding; active infection, active peptic ulcer, active cardiac disease requiring therapy, unstable diabetes mellitus; other documented malignancy except treated nonmelanoma skin cancer, carcinoma *in situ* of the cervix, or other cancers diagnosed at least 5 years previously and without recurrence. The study was conducted according to ICH Good Clinical Practice Guidelines, including obtaining informed consent from all patients.

A minimisation randomisation process (Pocock, 1983) was used in which patients were stratified on stage of disease (Okuda I or II), KPS (70–80 or 90–100) and whether they had prior intra-arterial chemotherapy (yes or no). A block size of 4 was used when there was balance between the prognostic factors and a ratio of 3 : 2 with a block size of 5 was used if there was imbalance. In both arms, gemcitabine was given at 1250 mg m^−2^ as an intravenous infusion on days 1 and 8 of 3-weekly cycles. In the standard schedule arm, gemcitabine was administered over 30 min, in the FDR arm at 10 mg m^−2^ min^−1^ (eg, over 125 min for a dose of 1250 mg m^−2^). An infusion pump was used to ensure exact infusion time. Antiemetics were used according to the standard local practice.

To start a next cycle, ANC had to be ⩾1.0 × 10^9^ l^−1^, platelets ⩾75 × 10^9^ l^−1^, AST, ALT and serum bilirubin ⩽4 × ULN, and patients had to have no other toxicity of common toxicity criteria (CTC) Grade 3 or 4 with the exception of nausea, vomiting and anaemia. The dose of gemcitabine was reduced by 20% in a subsequent cycle in the case of ANC <0.5 × 10^9^ l^−1^ associated with fever (defined as a single episode of ⩾38.5°C or three episodes of ⩾38.5°C during a 24-h period or lasting more than 7 days), platelet count <25 × 10^9^ l^−1^ or <50 × 10^9^ l^−1^ associated with bleeding, or AST, ALT or serum bilirubin >4 × ULN at any time during the preceding cycle. If any other toxicity of CTC Grade 3 or 4 occurred during the preceding cycle, with the exception of nausea, vomiting and anaemia, the dose of gemcitabine could be reduced by 20% at the discretion of the investigator. The day 8 dose of gemcitabine was omitted in case of ANC <0.5 × 10^9^ l^−1^, platelets <50 × 10^9^ l^−1^, AST, ALT or serum bilirubin >4 × ULN, or any other toxicity of CTC Grade 3 or 4 with the exception of nausea, vomiting and anaemia. Prophylactic use of granulocyte colony-stimulating factor (G-CSF) or granulocyte–macrophage colony-stimulating factor (GM-CSF) was not allowed in this study. Therapeutic CSF was permitted in case of febrile neutropaenia.

Before study enrolment, patients were required to have a physical examination, chest X-ray, abdominal and chest computed tomography, complete blood work-up and ECG. Abdominal ultrasound and bone X-ray and/or scan were optional as clinically indicated. Before the start of each cycle and day 8 dosing of gemcitabine, a physical examination, full blood count and blood work-up were performed. A full blood count was obtained around day 15. Upon discontinuation of study therapy, survival status was assessed until 12 months past randomisation or death, whichever occurred first. If patients had an objective remission or stable disease at the time of discontinuation of study therapy, response status was evaluated every 2 months.

Tumour response status was evaluated every two cycles. Confirmation of response was required at no earlier than 4 weeks. Complete response was defined as complete disappearance of all known disease; partial response as at least 50% reduction in the size of measurable lesions; no change as less than 50% reduction and 25% increase in the size of measurable lesions; and progressive disease as equal to or more than 25% increase in the size of at least one measurable lesion or any appearance of a new lesion. Duration of response and stable disease, and time to progression were measured from the date of randomisation to documented disease progression. Survival time was calculated from the date of randomisation to the date of death or last follow-up. Toxicity was graded according to the National Cancer Institute Common Toxicity Criteria, version 2.0 (National Cancer Institute, 1999).

The primary objective of this study was to evaluate the response rate of either treatment. One of the secondary objectives was to select the gemcitabine schedule with the higher therapeutic ratio (ie, activity *vs* toxicity) for further study in advanced HCC. Based on the data from Taiwan (Yang *et al*, 2000), a minimum response rate of 10% was assumed for either arm. The Simon design for randomised phase II studies was applied to select a treatment regimen for further investigation (Simon *et al*, 1985). Assuming a mean true response rate of 10%, and the true response rate achieved by one treatment to be 10% higher, a total sample size of 56 patients gave a probability of higher than 0.85 such that the better of the two treatments will have the higher observed response rate. Kaplan–Meier statistics were used to estimate time-to-event measures and log rank test was used for comparisons of treatment groups. Toxicity of the two arms was compared using Fisher's exact test.

## RESULTS

In total, 50 patients were entered on trial. Two patients did not receive study therapy because they did not meet the eligibility criteria. In all, 25 and 23 patients received gemcitabine at standard schedule and FDR, respectively. Patient enrolment was stopped early. Two reports of no activity by single-agent gemcitabine in advanced HCC (Kubicka *et al*, 2001; Ulrich-Pur *et al*, 2001) prompted an unplanned interim analysis, which found one partial response in 42 patients evaluable for response assessment. This level of activity was considered too low to warrant further patient enrolment.

All data reported here are based on the 48 patients who received study therapy. Of these, 26 patients were enrolled in the People's Republic of China, 15 in Thailand, six in South Korea and one in Hong Kong. The two treatment groups were well balanced for major baseline characteristics (Table 1

**Table 1:** Patient and tumour characteristics

	**Standard schedule (*n*=25)**	**FDR (*n*=23)**
*Age (years)*		
Median	48.7	49.0
Range	35.2–70.3	31.8–66.7
		
*KPS*		
Median	80	80
Range	70–100	70–90
		
*Stage*		
IIIB	4	2
IV	21	21
Two or more organ sites involved	5	12
		
*Okuda stage*		
I	12	9
II	13	14
		
*Prior therapy*		
None	22	20
Surgery	1	2
IACE	1	1
IAE	1	0
		
*History of chronic benign liver disease*		
None	6	9
Chronic hepatitis	12	9
Liver cirrhosis	7	5
		
*Hepatitis B serology*		
Positive	18	21
Negative	3	0
Unknown	4	2
		
*Hepatitis C serology*		
Positive	5	1
Negative	7	11
Unknown	13	11

IACE=intra-arterial chemo-embolisation; IAE=intra-arterial embolisation.

). There were 43 male and five female patients. In all, 12 patients had a tissue diagnosis of HCC, and in 36 patients the diagnosis was based on elevation of serum alpha-fetoprotein of higher than 400 ng ml^−1^ plus liver imaging studies considered as highly suggestive of HCC.

### Efficacy

**Figure 1:**
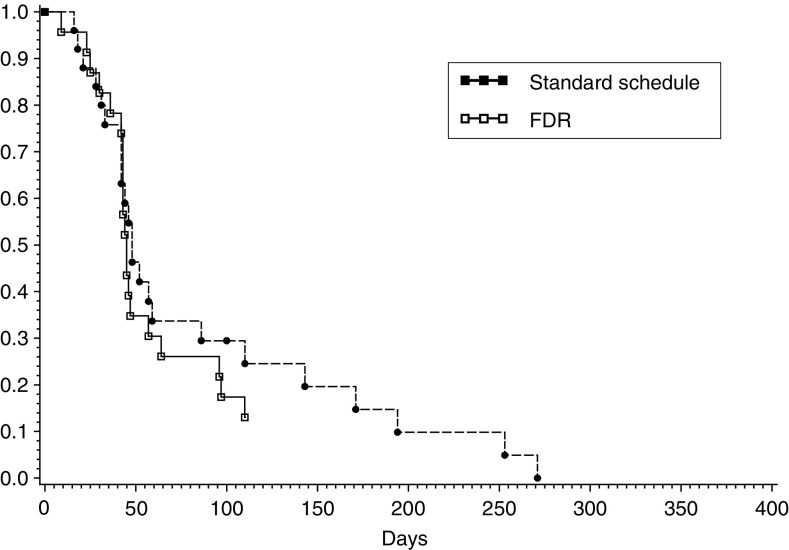
Time to progression by treatment arm.

No patient in the FDR arm and one patient in the standard schedule arm had an objective partial response, for an overall response rate of 2.1% (1 out of 48 patients; 95% CI: 0.05–11.1%). The response rates for the standard schedule and FDR arm were 4% (1 out of 25 patients; 95% CI: 0.1–20.4%) and 0% (0 out of 23 patients; 95% CI: 0–14.8%), respectively. The duration of the single partial response was 29.7 weeks. In total, 26 patients (54%) had progressive disease as best response. The time to progression and median survival time were 46 (95% CI: 43–57) and 97 (95% CI: 71–143) days, respectively, with no statistical differences between the two arms (Figures 1

and 2

**Figure 2:**
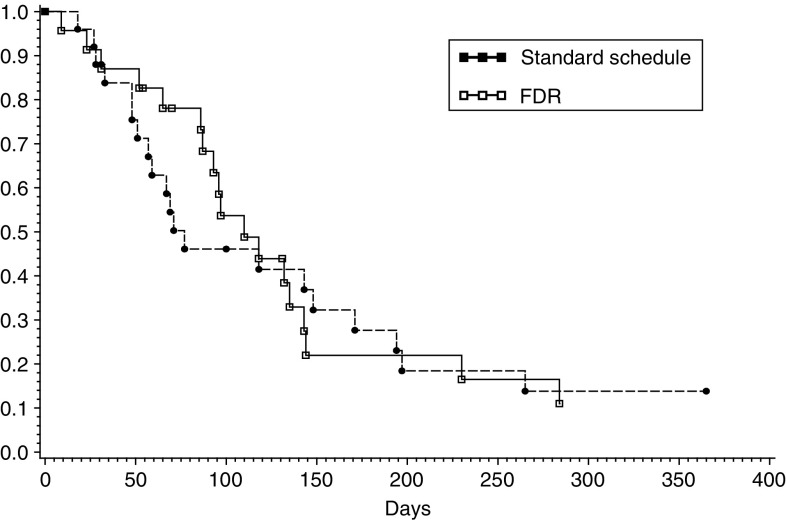
Survival by treatment arm.

). Five patients (11.6%) were alive at 1 year.

### Toxicity

In the standard schedule arm, the rates of Grade 3/4 haematological and nonhaematological toxicities were 44.0 and 76.0%, respectively. The corresponding rates in the FDR arm were 34.8 and 52.2%, respectively, with no statistical difference between the two treatment arms. Individual Grade 3/4 toxicities are listed in Table 2

**Table 2:** Grade 3 and 4 toxicities

	**Standard schedule (*n*=25)**		**FDR (*n*=23)**	
	**3**	**4**	**3**	**4**
Leukopaenia	2	0	3	0
Neutropaenia	4	1	2	2
Thrombocytopaenia	4	0	1	0
Anaemia	4	0	3	1
Febrile neutropaenia	0	0	0	0
Infection	0	0	1	0
Bleeding	3	1	0	0
AST/ALT	8	0	4	0
GGT	7	0	5	0
Bilirubin	1	0	2	0
Nausea	0	0	0	0
Vomiting	0	0	1	0
Skin rash/desquamation	1	0	0	0
Fatigue	3	0	1	1

. There were no drug-related deaths. Six patients (12.5%) received G-CSF in 8 out of 113 treatment cycles (7.1%). The number of cycles administered was 73 in the standard schedule and 40 in the FDR arm. The mean number of cycles per patient for the entire study population was 2.35, with a range of 1–6. Five patients completed six cycles of study therapy, all on the standard schedule arm. There were no signs of cumulative toxicity. The most common cause of early discontinuation was disease progression. Dose adjustments in subsequent cycles and on day 8 occurred in four (3.5%) and 36 (32%) cycles, respectively. With a planned dose intensity of 833 mg m^−2^ week^−1^ the actual dose intensity achieved was 703.4 mg m^−2^ week^−1^, for a relative dose intensity of 0.844. There was no significant difference in dose intensity between the two arms.

## DISCUSSION

Hepatocellular carcinoma is known to be highly resistant to chemotherapy (Fong *et al*, 2001). Doxorubicin is widely considered the most active single-agent, but more recent studies have failed to demonstrate meaningful activity (Lai *et al*, 1988, 1989; Mok *et al*, 1999). Newer agents such as raltitrexed, paclitaxel, irinotecan, nolatrexed and eniluracil-5-fluorouracil have shown no activity (Rougier *et al*, 1997; Chao *et al*, 1998; Mok *et al*, 1999; Stuart *et al*, 1999; Llovet *et al*, 2001; O'Reilly *et al*, 2001).

The present study did not show promising activity of single-agent gemcitabine. Preliminary data in advanced pancreatic cancer (Tempero *et al*, 1999) led us to hypothesise that gemcitabine administered at FDR may achieve higher activity in HCC. However, no objective remission was obtained in the FDR arm of this study. A phase II study of single-agent gemcitabine in Taiwan found a response rate of 17.8% in 28 patients with advanced HCC (Yang *et al*, 2000). More recently, three studies in Europe and the US have shown no activity (Kubicka *et al*, 2001; Ulrich-Pur *et al*, 2001; Fuchs *et al*, 2002). This discrepancy may suggest differing sensitivity of HCC in Asia *vs* Western countries. However, the present trial was conducted in three Asian countries and was not able to reproduce the activity reported from Taiwan. The eligibility criteria in the current and Taiwan trial were similar, and so were the reported characteristics of the study patients. However, the median survival in the Taiwan study was 18.7 weeks, while it was 13.9 weeks (97 days) in the present study. This suggests more favourable prognostic features of the patients in the Taiwan study, which may also have increased the probability of tumour response. However, the median survival in the US study was 6.9 months and no objective response was observed (Fuchs *et al*, 2002). The median survival of 97 days in the current trial is similar to the 3.1 months found for HCC patients classified as intermediate-risk by the Chinese University Prognostic Index (CUPI) (Leung *et al*, 2002). We performed a retrospective risk classification of the study patients according to the CUPI and found 19 (39.6%) and 28 (58.3%) patients falling in the intermediate- and low-risk categories, respectively. The mean CUPI for the overall population was +1.33 (range, −4 to +9), which is close to the lower CUPI cutoff value of +2 for intermediate risk (Leung *et al*, 2002). Accordingly, the survival outcome in the present study seems to be consistent with the Hong Kong study (Leung *et al*, 2002).

Gemcitabine was used at full dose, yet toxicity was well manageable, with a low rate of Grade 3 or 4 clinical toxicities, low need for G-CSF, and no drug-related deaths. The relative dose intensity was 0.844. All other studies of gemcitabine in HCC, used as single agent or in combination with cisplatin or doxorubicin, have shown mild to modest toxicity (Babu *et al*, 2000; Yang *et al*, 2000, 2002; Kubicka *et al*, 2001; Ulrich-Pur *et al*, 2001; Fuchs *et al*, 2002). We anticipated higher toxicity for FDR gemcitabine, as has been previously observed (Brand *et al*, 1997; Tempero *et al*, 1999). Interestingly, this was not the case. As is common in HCC, many patients in this study had underlying benign chronic liver disease. In Asia, this is typically the result of hepatitis B infection, as was the case in the present study. Of note, no hepatitis flare was observed.

In conclusion, both schedules of gemcitabine were found to be safe and toxicity was well manageable in this patient population, but gemcitabine seems no more active than other cytotoxic agents when used alone for systemic treatment of advanced HCC.
